# Changes in Immunoreactivity of Sensory Substances within the Enteric Nervous System of the Porcine Stomach during Experimentally Induced Diabetes

**DOI:** 10.1155/2018/4735659

**Published:** 2018-07-24

**Authors:** Michał Bulc, Katarzyna Palus, Jarosław Całka, Łukasz Zielonka

**Affiliations:** ^1^Department of Clinical Physiology Faculty of Veterinary Medicine, University of Warmia and Mazury in Olsztyn, Oczapowskiego Str. 13, 10-719 Olsztyn, Poland; ^2^Department of Veterinary Prevention and Feed Hygiene, Faculty of Veterinary Medicine, University of Warmia and Mazury in Olsztyn, 10-718 Olsztyn, Poland

## Abstract

One of the most frequently reported disorders associated with diabetes is gastrointestinal (GI) disturbance. Although pathogenesis of these complications is multifactorial, the complicity of the enteric nervous system (ENS) in this respect has significant importance. Therefore, this paper analysed changes in substance P- (SP-), calcitonin gene-related peptide- (CGRP-), and leu5-enkephalin- (L-ENK-) like immunoreactivity (LI) in enteric stomach neurons caused by chemically induced diabetes in a porcine model. Using double immunofluorescent labelling, it was found that acute hyperglycaemia led to significant changes in the chemical coding of stomach enteric neurons. Generally, the response to artificially inducted diabetes depended on the “kind” of enteric plexus as well as the stomach region studied. A clear increase in the percentage of neurons immunoreactive to SP and CGRP was visible in the myenteric plexus (MP) in the antrum, corpus, and pylorus as well as in the submucosal plexus (SmP) in the corpus. For L-ENK, an increase in the number of L-ENK-LI neurons was observed in the MP of the antrum and SmP in the corpus, while in the MP of the corpus and pylorus, a decrease in the percentage of L-ENK-LI neurons was noted.

## 1. Introduction

Diabetes mellitus is actually considered an epidemic in the 21st century. The diabetic population has systematically increased worldwide, and it is expected to rise to 592 million patients/cases by the year 2035 [1]. Improper treatment of diabetes leads to long-term increased blood glucose level, which is considered to be a major pathophysiological factor in the development of severe diabetic complications [2, 3]. It has been evaluated that up to 75% of patients with type 1 or type 2 diabetes will have the complication of neuropathy. One of the clinical signs of neuropathy is gastrointestinal (GI) disorder [4–6]. Regardless of their etiology, it is possible that the molecular mechanisms of pathological changes within the GI tract are partly due to changes within the enteric nervous system (ENS) [4].

The enteric nervous system regulates gastrointestinal functions, which is independent of the central nervous system and is embedded in the wall of the gastrointestinal tract [7]. The ENS is comprised approximately of millions of sensory, motor, and interneurons formed in intramural ganglionated plexuses interconnected with a very dense network of nerves [8]. In the porcine stomach, it is formed by the myenteric plexus (MP) located between the longitudinal and circular muscle layers, which mainly regulates the proper gastric motility, both after and between the meals, and the submucosal plexus (SmP) is situated in the inner side of the circular muscle layer, primarily regulating fluid secretion and resorption [9, 10].

Gastrointestinal complications in the course of diabetes mellitus appear to be multifactorial [11]. Several possibilities have been suggested, such as diabetic autonomic neuropathy altering the parasympathetic and sympathetic nerve functions. Moreover, other possibilities have been proposed. Namely, enteric neurons may undergo changes caused by adaptive and/or neuroprotective processes during many gastrointestinal and extraintestinal diseases, and these changes are mainly manifested in the modification of neuronal chemical phenotyping [12, 13]. Proper sensory perception is conditioned by a functional balance between nociceptive substances such as substance P (SP) or calcitonin gene-related peptide (CGRP) and, on the other hand, an appropriate level of antinociceptive factors, particularly the opioid family, for example, leu5-enkephalin (L-ENK) [14–17].

SP is an undecapeptide and belongs to the tachykinin/neurokinin family. The neurokinin family consists of (in addition to SP) neurokinin A (NKA) and neurokinin B (NKB) [18]. SP and NKA are encoded by the preprotachykinin I gene, while NKB is encoded by the preprotachykinin II gene [19]. SP is widely distributed in the ENS. Till now, SP-like immunoreactivity has been described in different fragments of the GI tract, like the oesophagus, stomach, duodenum, jejunum, and descending colon [20]. SP in the GI tract is first of all considered to be a neurotransmitter for primary sensory afferent nerve fibres, which are involved in pain signal transmission. Furthermore, SP is engaged in the regulation of blood flow, both by relaxation and by contraction of muscular cells of blood vessels [21–24]. The expression of SP within the enteric nervous system, as well as their changes, has been described in various species under different pathological conditions, including inflammatory bowel disease [22], bisfenol A intoxication [25], parasite infection [26], *Bacteroides fragilis* infection [27], or carcinoma [28].

Calcitonin gene-related peptide (CGRP) is a member of the calcitonin family of peptides [29]. CGRP is one of the most abundant peptides, produced in both the peripheral and central nervous systems [28]. One of the better-known functions of CGRP in the GI tract is its participation in nociception signal transmission. It is also known that CGRP plays a role as a neuromediator and/or neuromodulator, participating in the regulation of motor activity, blood flow, and gastric acid secretion [27]. CGRP is also considered to be a marker of intrinsic primary afferent neurons. This class of neurons plays an essential role in short reflexes in the intestinal wall excluding the central nervous system. This mentioned function corresponds with wide distribution of CGRP in the GI tract. Namely, CGRP-immunoreactive nerve structures were described in the wall of the GI tract from the oesophagus to the rectum [30]. Multiple functions of CGRP in the GI tract were confirmed by numerous studies regarding pathological conditions resulting in changes in expression of CGRP [30].

In turn, met-enkephalin, leu-enkephalin, *β*-endorphin, *α*-neo-endorphin, dynorphin, and nociceptin/orphanin are opioid peptides. Leu-enkephalin (L-ENK) was the first time isolated from the porcine brain in 1975. Opioid peptides in the stomach originate from both enteroendocrine cells and ENS neurons. LENK is a factor that most often is present in sensory neurons and its participation in sensory and pain transmission is well known [31, 32].

Therefore, this experiment was designed to determine the possible alterations in expression of sensory substances in the stomach enteric neurons under the influence of acute hyperglycaemia. Streptozotocin-induced hyperglycaemic/diabetic porcine model was used in this study. In many publications on diabetic complications, much attention is paid to issues that affect life-threatening conditions or are threatened with irreversible crippling. As high as 75% of patients with diabetes may experience gastrointestinal symptoms. These complications represent a major cause of morbidity and have a negative impact on healthcare (leads to a significant impairment in the quality of life) and costs in diabetes. Taking this into account, it seems highly important to find an appropriate animal model that allows learning the exact etiology of changes occurring in diabetes in the gastrointestinal tract. Especially in the area of diabetes, the swine in the last time has gained considerable interest [33, 34]. It is due to the fact that biochemical and pathophysiological responses to diabetes remain in part like those observed in people. It has been documented that blood flow of the pig pancreas is similar to that of the human pancreas and the number of insulin producing cells is within a similar number as that observed in humans [35]. Moreover, pigs, in contrast to rodents, are omnivorous and active during the day. It all makes the pig a much better model for studying diabetic complications than rodents. The current study for the first time focused on the examination of SP, CGRP, and leu5-enkephalin expression in stomach neurons during streptozotocin-inducted diabetes. The choice of these neuropeptides was dictated by the fact that these substances are involved in sensory signal modulation. It is well documented that abdominal pain is one of the most serious GI diabetic complications. The current results may help to create the foundation for further, more detailed, and clinically oriented studies, involving these neuropeptides and their roles in gastrointestinal diabetic symptom treatment.

## 2. Materials and Methods

### 2.1. Animals

We followed the methods of Bulc et al. [36]. This study was conducted on ten juvenile female pigs of the white large Polish breed, weighing from 17.0 kg to 20 kg. At the beginning of the experiment, the animals were randomly distributed into two groups: the diabetic group (D, *n* = 5) and the control group (C, *n* = 5) and were housed in cages suitable for pigs. The animals were given one week of acclimatization to observe their general health, to minimize physiological stress, and to ensure the proper conduct of the study. The treatment of animals was conducted in compliance with the instructions of the Local Ethical Committee in Olsztyn (Poland) (decision number 13/2015/DTN) with special attention paid to minimizing any stress reaction.

### 2.2. Chemical Induction of Diabetes

After acclimatization, hyperglycaemia was induced as previously described [36]. Streptozotocin (STZ) (Sigma-Aldrich, St Louis, MO, USA, S0130), 150 mg/kg of body weight, was dissolved in a freshly prepared disodium citrate buffer solution (pH = 4.23, 1 g streptozotocin/10 mL solution). For this purpose, pigs were anesthetized and the solution was administrated via an intravenous needle inserted into an ear with continuous infusion for approximately 5 minutes. To avoid gastrointestinal complications such as nausea and vomiting after streptozotocin injection, animals were fasted for 18 h before the experiment and the control pigs were injected with equal amounts of vehicle (citrate buffer).

The pigs were continuously observed for 24 h after streptozotocin injection. Because streptozotocin often causes temporary hypoglycaemia, 250 mL of 50% glucose solution per animal was administered. The pigs received a normal diet throughout the experiment twice a day and tap water ad libitum. The blood glucose level was measured to confirm hyperglycaemia. The blood glucose concentration was estimated using an Accent-200 (Germany) biochemical analyser, with the colorimetric measurement at a wavelength of 510 nm/670 nm. For this purpose, capillary blood from the ear was collected. The plasma glucose level was measured prior to the experiment initiation in both control and experimental groups. The next measurement was made 48 hours after the induction of diabetes. Subsequent measurements of glucose levels were monitored weekly until the end of the experiment.

### 2.3. Tissue Collection

Six weeks after streptozotocin injection, pigs were deeply anesthetized via intravenous administration of pentobarbital (Vetbutal, Biowet, Poland) and perfused transcardially via the ascending aorta with 4% paraformaldehyde in a 0.1 M phosphate buffer (PB, pH 7.4). The samples were postfixed by immersion in the same fixative for 1 h, rinsed several times with (PB), and then transferred into a 30% sucrose solution and stored at 4°C until sectioning. The frozen tissue blocks were cut in frontal or sagittal planes using a Microm HM 560 cryostat (Carl Zeiss, Germany) at a thickness of 12 *μ*m and mounted on chrome-coated slides.

### 2.4. Immunofluorescence Procedure

The sections were processed for double immunofluorescence staining. Briefly, after air drying at room temperature for 45 min and rinsing in 0.1 M phosphate-buffered saline (PBS; pH 7.4; 3 × 10 min), the sections were incubated in a blocking buffer containing 10% normal goat serum (MP Biomedicals, USA), in 0.1 M PBS, 0.1% donkey serum (Abcam, UK), 1% Triton X-100 (Sigma-Aldrich, USA), 0.05% Thimerosal (Sigma-Aldrich, USA), and 0.01% NaN_3_ for 1 h at room temperature to reduce nonspecific background staining. Subsequently, after another wash in PBS (3 × 10 min), the sections were incubated overnight at 4°C with primary antibodies raised in different species and directed towards general neuronal marker Hu C/D proteins (mouse polyclonal: Invitrogen USA; code A-212711:1.000; working dilution 1 : 1000), anti-nSP antibodies (rat monoclonal: AbD Serotec, cat. number 8450-0505; working dilution 1 : 150), CGRP (rabbit polyclonal; Millipore, cat. number AB15360; working dilution 1: 4000), and L-ENK (rabbit polyclonal: Abcam, cat. number ab85798; working dilution 1 : 1000). All antibodies were diluted in PBS containing 0.3% Triton X-100 and 1% BSA. On the following day, the sections were rinsed (PBS, 3 × 15 min) and incubated with secondary antibodies (donkey anti-mouse Alexa Fluor 488, 1 : 1000 Invitrogen USA, code A21202; donkey anti-rabbit Alexa Fluor 546 1 : 1000 Invitrogen, USA, code A10040 diluted in PBS containing 0.25% BSA and 0.1% Triton X-100; and donkey anti-rat Alexa Fluor 546, 1 : 1000 Invitrogen, USA; code A21208 diluted in PBS containing 0.25% BSA and 0.1% Triton X-100) for 4 hours. The sections were then rinsed three times (PBS, 3 × 5 min) and mounted in fluorescent mounting medium (DAKO, Carpinteria, CA, USA). The prepared specimens were viewed and photographed using an Olympus BX51 microscope equipped with epifluorescence and appropriate filter sets, coupled with a digital monochromatic camera (Olympus XM 10) connected to a PC, and analysed with Cell Dimension software (Olympus, Tokyo, Japan). Standard controls, that is, preabsorption of the neuropeptide antisera with appropriate antigen, omission, and replacement of the primary antisera by nonimmune sera, were performed to test the antibodies and specificity of the method. The test was performed as follows: sections of the stomach were incubated with a “working” dilution of the primary immunoserum, which had been previously preabsorbed for 18 h at 37°C with 20 *μ*g of appropriate purified protein SP (ab120170, Abcam), CGRP (ab158017, Abcam), and L-ENK (ab159087, Abcam). Additional negative controls, such as the omission and replacement of all primary antisera with nonimmune sera, were also performed. This procedure completely eliminated specific staining.

### 2.5. Counting of the Nerve Structures and Statistical Evaluation

The number of SP-, CGRP-, and L-ENK-like immunoreactive (LI) enteric neurons was expressed as a percentage of the total number of Hu C/D-positive perikarya. At least 700 Hu C/D-labelled cell bodies of intramural ganglia of each part of the stomach were examined. Only neurons with well-visible nucleus were counted. To prevent the double counting of Hu C/D-immunoreactive neurons, the sections were located at least 100 *μ*m apart. The data pooled from all animal groups were statistically analysed using Statistica 10 software (StatSoft Inc., Tulsa, OK, USA) and expressed as a mean ± standard error (SE) of mean. Significant differences were evaluated using Student's *t*-test for independent samples (^∗^*P* < 0.05, ^∗∗^*P* < 0.001, and ^∗∗∗^*P* > 0.001).

Evaluation of the density of nerves within the muscular or mucosal layers was based on counting all the nerve fibres which were immunoreactive to SP, CGRP, and L-ENK per microscopic observation and was assessed under ×40 objective (0.55 mm2) by subjective observation (two independent researchers). Nerve profiles with clearly visible varicosities were counted in four sections per animal (in five fields per section) and the obtained data were pooled and presented as a mean ± SEM.

## 3. Results

**Figure 1:**
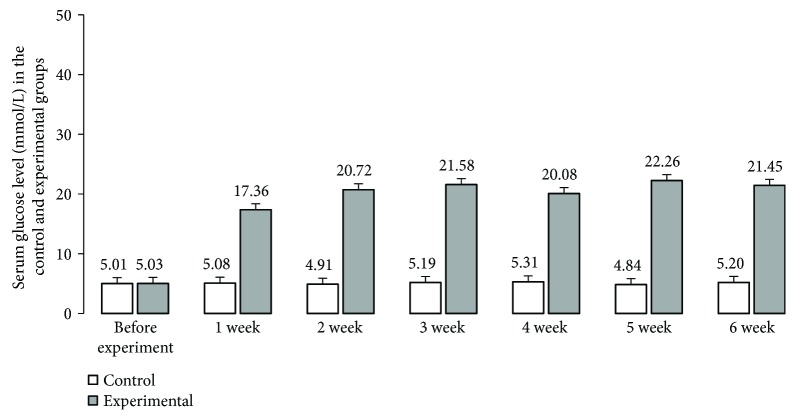
Serum glucose levels. Serum glucose levels after induction of diabetes and glucose concentration after streptozotocin administration (1 to 6 weeks).

The mean glycaemia level in pigs before streptozotocin injection was within standard reference values for the pig (5.01 mmol/L ± 0.10 mmol/L) (Figure 1) and did not differ between individual animals. Following the STZ induction, a consistent increase in plasma glucose concentration was observed (Figure 1). A significant (17.36 mmol/L ± 0.38 mmol/L) increase in glucose level was observed on the 7th day after STZ injection. The highest increase in blood glucose concentration was detected 4-5 weeks after the STZ injection (22.26 mmol/L ± 1.21 mmol/L) (Figure 1). In the last week of the experiment, the mean serum glucose level increased slightly, reaching 21.24 mmol/L ± 1.11 mmol/L (Figure 1). It should be noted that although chronic glycaemia in experimental animals was significantly higher than that in controls, all pigs which received streptozotocin survived the duration of the experiment in a good general condition and none of the animals required exogenous insulin injection.

**Figure 2:**
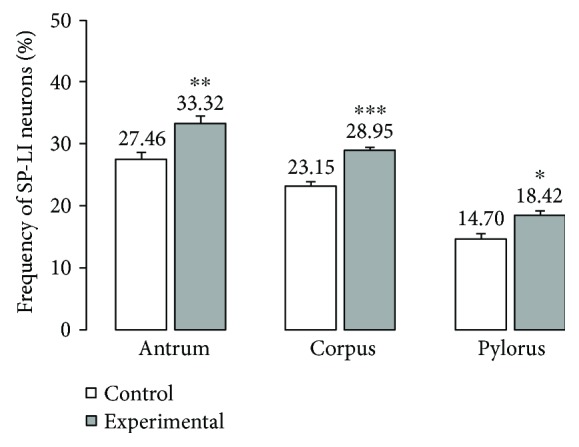
The average percentage of MP neurons immunoreactive to SP. The mean of SP-like immunoreactive (SP-LI) neurons in the myenteric plexus (MP) of the stomach antrum, corpus, and pylorus regions in the control and streptozotocin-inducted diabetes groups. Data are presented as mean ± SEM; statistically significant data (^∗^*P* < 0.05, ^∗∗^*P* < 0.01, and ^∗∗∗^*P* < 0.001).

**Figure 3:**
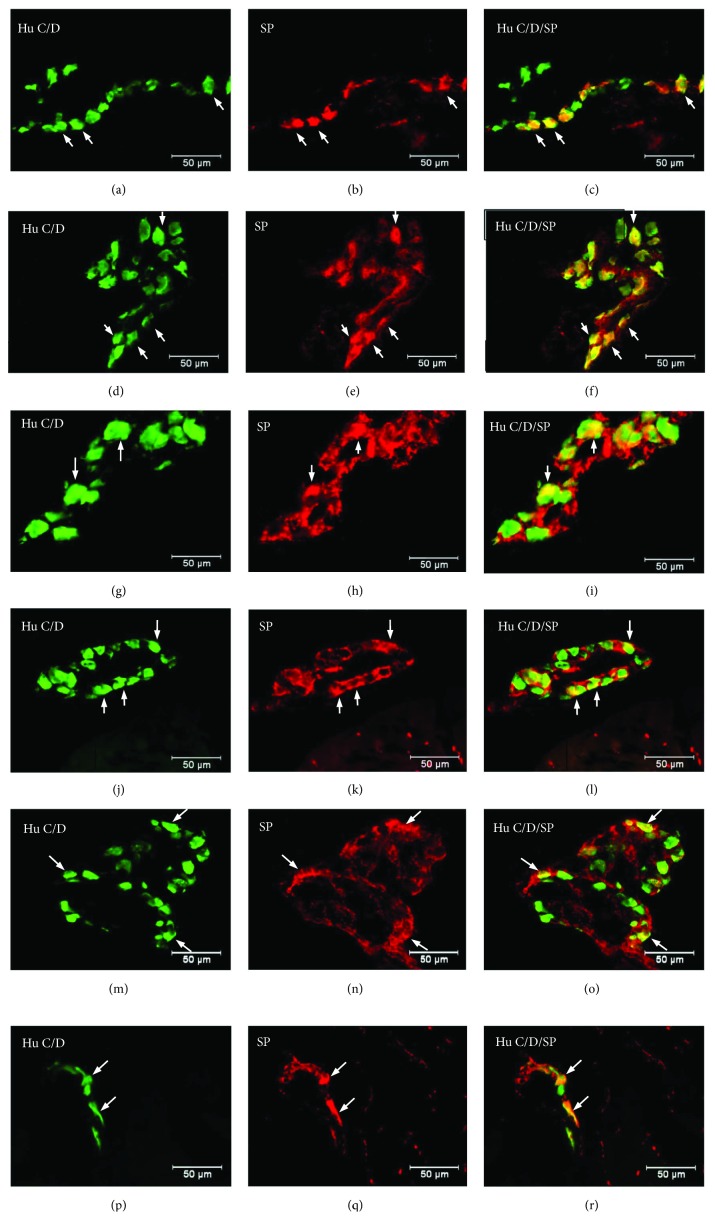
Myenteric ganglion of the porcine stomach immunoreactive to SP. Myenteric ganglion of the porcine antrum under physiological condition (a–c) and during experimentally inducted diabetes (d–f); myenteric ganglion of the porcine corpus under physiological condition (g–i) and during experimentally inducted diabetes (j–l); and myenteric ganglion of the porcine pylorus under physiological condition (m–o) and during experimentally inducted diabetes (p–r), immunostained for Hu C/D (green/arrows) and SP (red/arrows). The right column of the pictures shows the overlap of both stainings. Colocalization of both antigens in the studied cell bodies is indicated with arrows.

**Figure 4:**
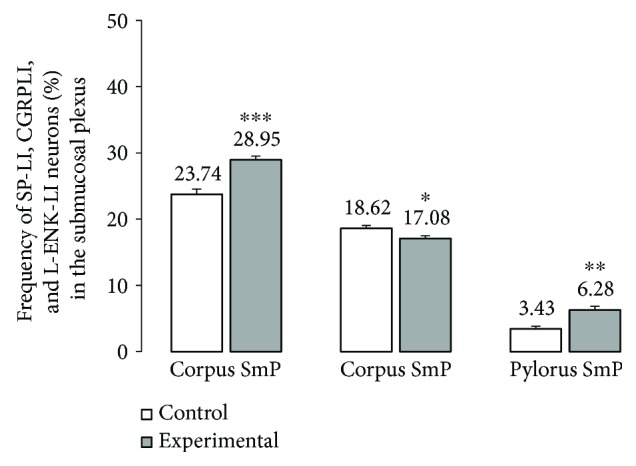
The average percentage of SmP neurons immunoreactive to SP, CGRP, and L-ENK. The mean of SP-like immunoreactive (SP-IL), CGRP-like immunoreactive (CGRP-LI), and L-ENK-like immunoreactive (LENK-LI) neurons in the submucosal plexus (SmP) of the stomach corpus in the control and streptozotocin-inducted diabetes groups. Data are presented as mean ± SEM; statistically significant data (^∗^*P* < 0.05, ^∗∗^*P* < 0.01, and ^∗∗∗^*P* < 0.001).

**Figure 5:**
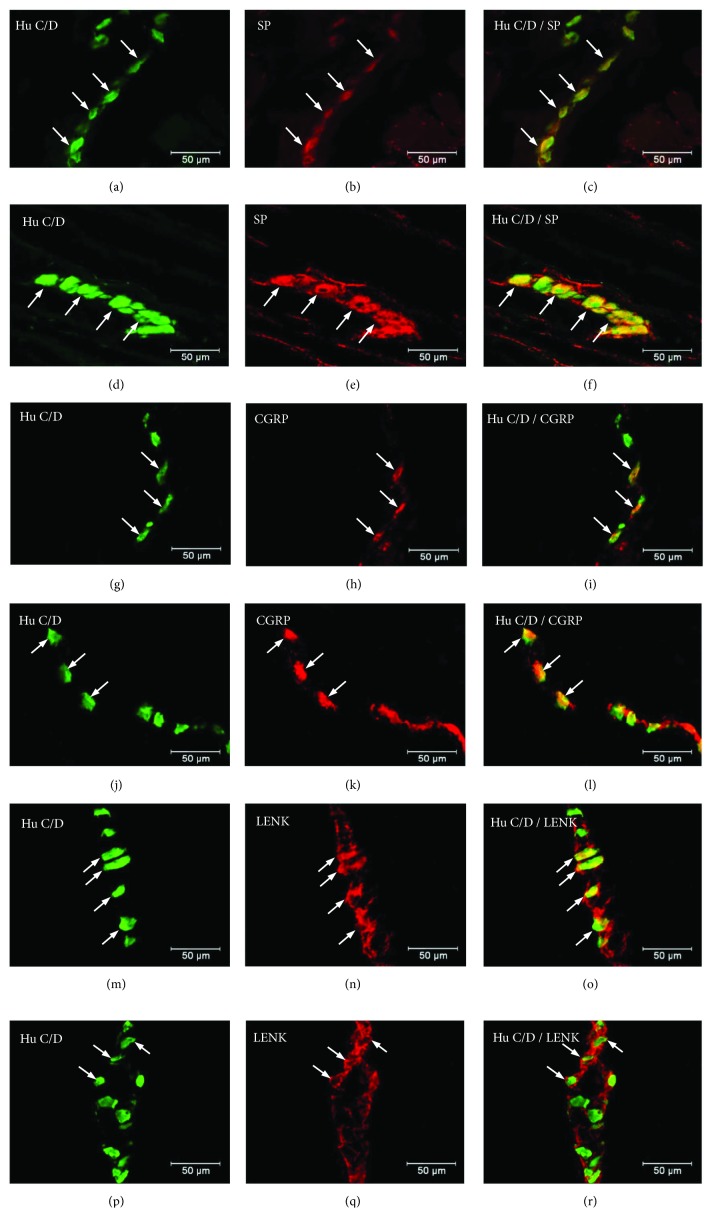
Submucosal ganglion of the porcine stomach immunoreactive to SP, CGRP, and L-ENK. Submucosal ganglion of the porcine corpus under physiological condition (a–c) and during experimentally inducted diabetes (d–f) immunoreactive to SP; submucosal ganglion of the porcine corpus under physiological condition (g–i) and during experimentally inducted diabetes (j–l) immunoreactive to CGRP; and submucosal ganglion of the porcine corpus under physiological condition (m–o) and during experimentally inducted diabetes (p–r) immunoreactive to L-ENK, immunostained for Hu C/D (green/arrows) and SP,CGRP, and L-ENK (red/arrows). The right column of the pictures shows the overlap of both stainings. Colocalization of both antigens in the studied cell bodies is indicated with arrows.

Double labelling immunohistochemistry revealed that in the control group, the SP distribution in the ENS neurons was varied and clearly depended on the analysed area of the stomach (Figure 2). In the myenteric ganglia of the antrum, the SP-positive cell bodies constitute 27.46 ± 1.09% of all HuC/D neurons studied (Figure 2, Figures 3(a)–3(c)). In turn, inside the MP of the corpus, the quantity of SP-positive cell bodies was slightly lower (23.15 ± 0.75%) (Figure 2, Figures 3(g)–3(i)). A very similar number of SP-LI neurons were observed in the submucosal plexus (SmP) of the corpus (23.74 ± 0.80%) (Figure 4, Figures 5(a)–5(c)). The smallest number of SP-LI perikarya was found in the MG of the pylorus (14.70 ± 0.80%) (Figure 2, Figures 3(m)–3(o)).

In the diabetic group, a statistically significant increase in the number of SP-positive cell bodies was observed in all investigated area of the stomach. In the MG of the antrum, the population of SP neurons amounted to 33.32 ± 1.21%. (Figure 2, Figures 3(d)–3(f)). The highest increase in SP expression was observed in the MG of the corpus, where the number of SP-LI neurons was 36.58 ± 0.64% (Figure 2, Figures 3(j)–3(l)). A slightly lower increase in SP-positive neurons (to 28.5 ± 0.56%) in the submucosal ganglia of the corpus was noted (Figure 4, Figures 5(d)–5(f)). With regard to the MG in the pylorus, changes in chemical coding were relativity smaller and amounted to 18.42 ± 0.82% (Figure 2, Figures 3(p)–3(r)).

**Figure 6:**
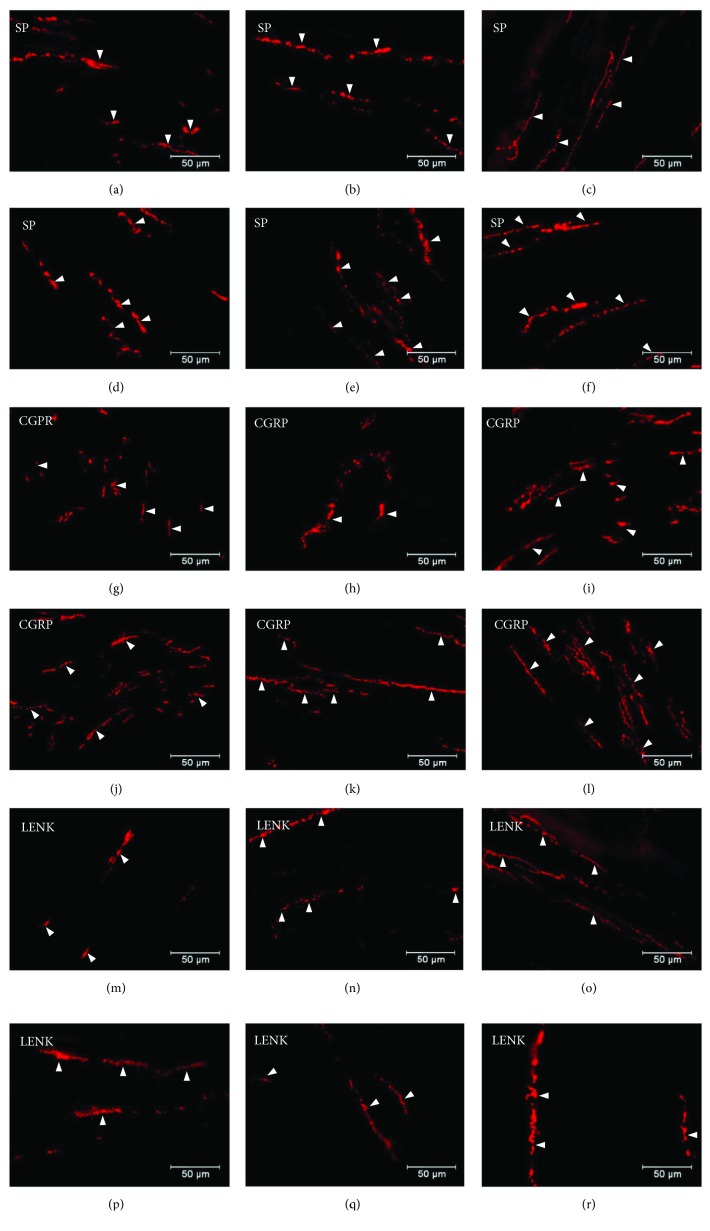
Distribution pattern of nerve fibres (arrow) immunoreactive to SP, CGRP, and L-ENK within the circular muscle layer. Distribution pattern of nerve fibres (arrows) within the circular muscle layer immunoreactive to SP under physiological condition: antrum (a), corpus (b), and pylorus (c) and under experimentally inducted diabetes: antrum (d), corpus (e), and pylorus (f); for CGRP under physiological condition: antrum (g) corpus (e), and pylorus (f) and under experimentally inducted diabetes: antrum (j), corpus (k), and pylorus (l); and for L-ENK under physiological condition: antrum (m), corpus (n), and pylorus (o) and under experimentally inducted diabetes: antrum (p), corpus (q), and pylorus (r).

**Figure 7:**
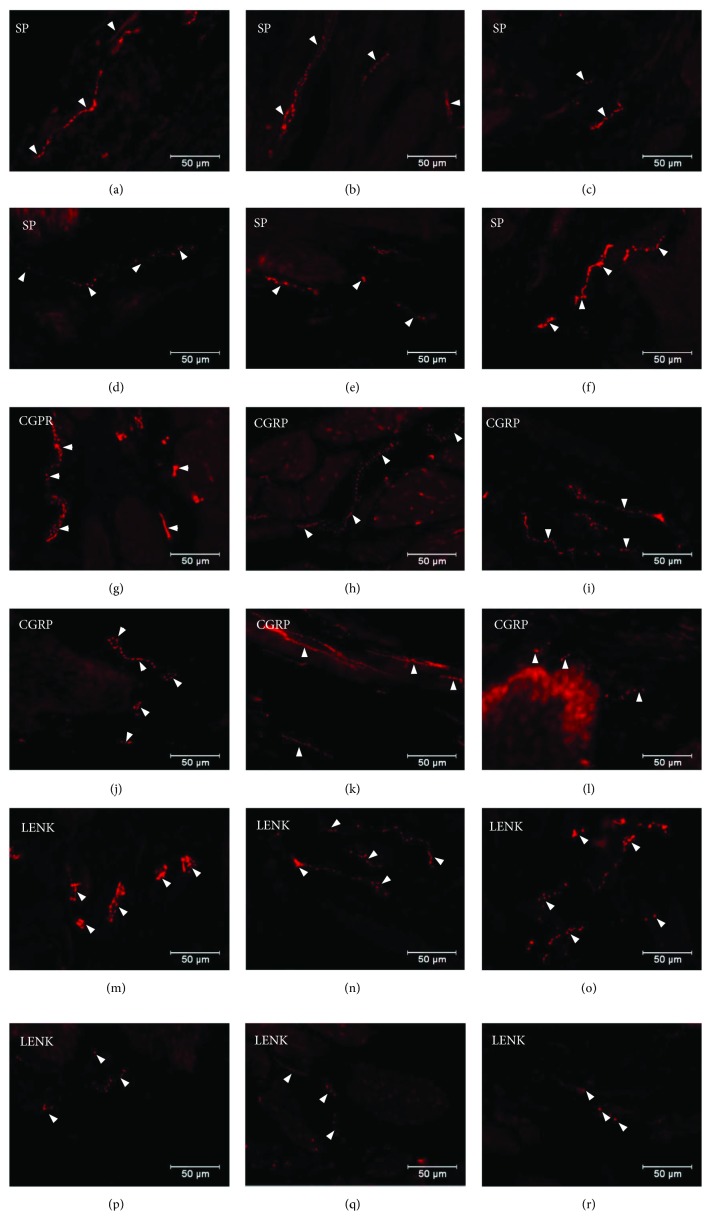
Distribution pattern of nerve fibres (arrow) immunoreactive to SP, CGRP, and L-ENK within the submucosal muscle layer. Distribution pattern of nerve fibres (arrows) within the submucosal muscle layer immunoreactive to SP under physiological condition: antrum (a), corpus (b), and pylorus (c) and under experimentally inducted diabetes: antrum (d), corpus (e), and pylorus (f); for CGRP under physiological condition: antrum (g), corpus (e), and pylorus (f) and under experimentally inducted diabetes: antrum (j), corpus (k), and pylorus (l); and for L-ENK under physiological condition: antrum (m), corpus (n), and pylorus (o) and under experimentally inducted diabetes: antrum (p), corpus (q), and pylorus (r).

Moreover, in addition to cell bodies, the density of nerve fibres was also investigated. Namely, SP-positive nerve fibres were present in the circular and submucosal muscle layers. In the control group, the SP-immunoreactive nerve fibres were more numerous in the circular muscle layer and they constitute 4.82 ± 0.16 in the antrum, 3.65 ± 0.16 in the corpus, and 6.07 ± 0.13 in the pylorus (Table 1, Figures 6(a)–6(c)). In the diabetic group, a statistically significant increase was observed in the density of SP-LI nerve fibres in the circular muscle layer in all areas of the stomach. In the antrum, SP-positive nerve fibres constituted 7.42 ± 0.37, 5.63 ± 0.16 in the corpus, and 10.75 ± 0.40 in the pylorus (Table 1, Figures 6(d)–6(f)). In the submucosal layer, the SP-positive nerve fibres in the control group were more numerous in the corpus 5.19 ± 0.20, while in the antrum and pylorus, they constituted only 2.8 ± 0.28 and 2.41 ± 0.18, respectively (Table 1, Figures 7(a)–7(c)). Under hyperglycaemia conditions, a statistically significant increase in the density of SP nerve fibres in the antrum and corpus was observed (6.72 ± 0.16 and 9.46 ± 0.41, resp.) (Table 1, Figures 7(d) and 7(e)), while in the pylorus, no statistically significant changes were observed (Table 1, Figure 7(f)).

**Figure 8:**
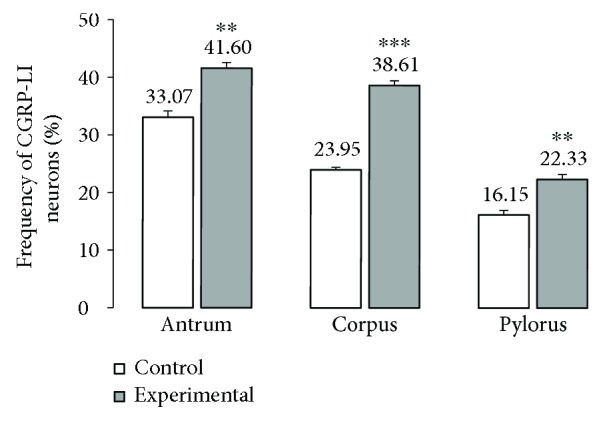
The average percentage of MP neurons immunoreactive to CGRP. The mean of CGRP-like immunoreactive (CGRP-LI) neurons in the myenteric plexus (MP) of the antrum, corpus, and pylorus regions of the stomach in the control and streptozotocin-inducted diabetes groups. Data are presented as mean ± SEM; statistically significant data (^∗∗^*P* < 0.01 and ^∗∗∗^*P* < 0.001).

**Figure 9:**
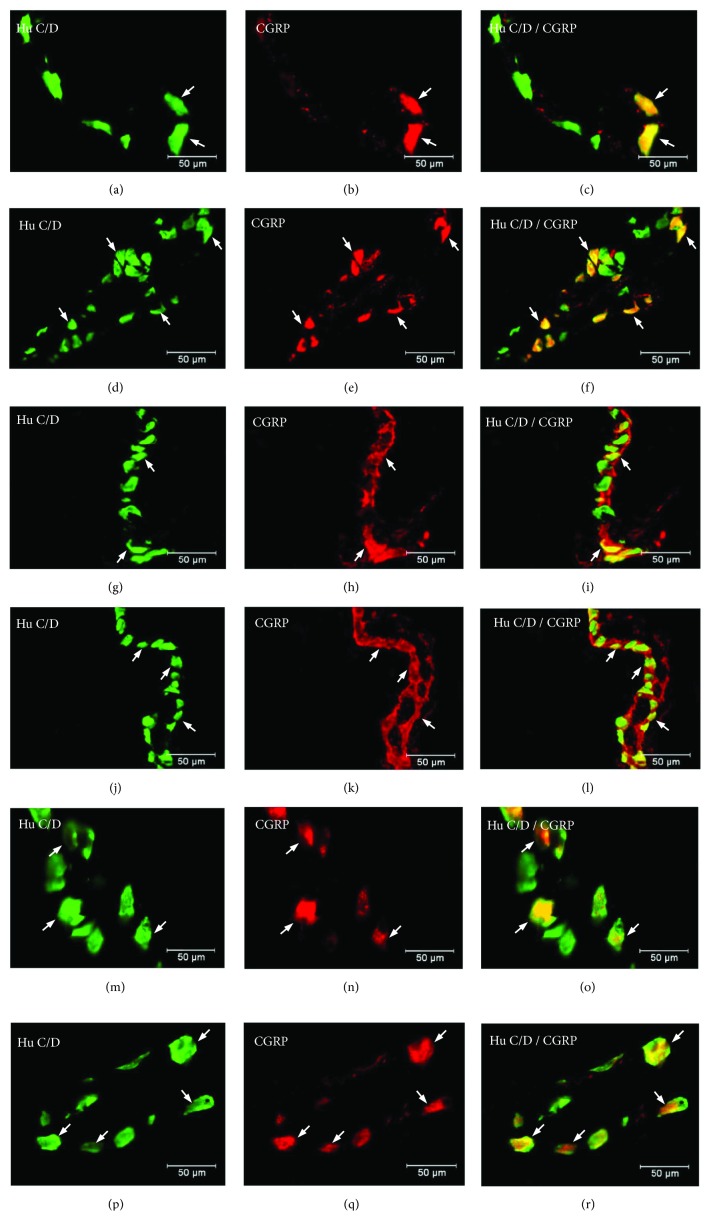
Myenteric ganglion of the porcine stomach immunoreactive to CGRP. Myenteric ganglion of the porcine antrum under physiological condition (a–c) and during experimentally inducted diabetes (d–f); myenteric ganglion of the porcine corpus under physiological condition (g–i) and during experimentally inducted diabetes (j–l); and myenteric ganglion of the porcine pylorus under physiological condition (m–o) and during experimentally inducted diabetes (p–r), immunostained for Hu C/D (green/arrows) and CGRP (red/arrows). The right column of the pictures shows the overlap of both stainings. Colocalization of both antigens in the studied cell bodies is indicated with arrows.

The other investigated substance was CGRP. Similar to SP, CGRP-positive neurons were presented in all studied areas, but clear differences were noted between various regions of the stomach. In the control group, the highest population of CGRP was noted in the antrum (33.07 ± 1.10%) (Figure 8, Figures 9(a)–9(c)), while in the other regions of the stomach, the number of CGRP-immunoreactive cell bodies was relatively lower and amounted to 23.95 ± 0.64% within the MG in the corpus (Figure 8, Figures 9(g)–9(i)) and 16.15 ± 0.80% in the pylorus (Figure 8, Figures 9(m)–9(o)) and 18.62 ± 0.44% in the submucosal ganglia in the corpus (Figure 4, Figures 5(g)–5(h)). In the diabetic group, statistically significant changes were observed in all investigated areas. The number of CGRP-positive neurons within the MG was estimated at 41.6 ± 0.99% in the antrum (Figure 8, Figures 9(d)–9(f)), 38.61 ± 0.81% in the corpus (Figure 8, Figures 9(j)–9(l)), and 22.33 ± 0.86% in the pylorus (Figure 8, Figures 9(p)–9(r)). In turn, in the submucosal ganglia inside the corpus, the numbers of GGRP-positive neurons were 17.08 ± 0.40% (Figure 4, Figures 5(j)–5(l)). Nerve fibres immunoreactive to CGRP were observed in the circular muscle layer and in the submucosal layer (Table 1). In the control group, the density of CGRP-LI neuronal processes was similar in all investigated areas. In the circular muscle layer, an average of 6.10 ± 0.37 of CGRP-positive fibres were noted in the antrum (Figure 6(g)), 6.20 ± 0.18 in the corpus (Figure 6(h)), and 6.24 ± 0.33 in the pylorus (Figure 6(i), Table 1).

In the diabetic group, a statistically significant increase in the density of CGRP-LI fibres within the circular muscle layer was observed. In the antrum, the density of CGRP-containing nerve fibres was estimated at 10.44 ± 0.23 (Figure 7(j)), whereas in the corpus and pylorus, it was 11.38 ± 0.32 (Figure 7(k)) and 8.85 ± 0.19, respectively (Table 1, Figure 7(l)). Within the submucosal layer, the density of CGRP-positive nerve fibres was scarce. In the antrum of control animals, 2.58 ± 0.12 of CGRP-positive nerve fibres was observed, while in the corpus, it was 2.08 ± 0.14 and it was 3.40 ± 0.18 in the pylorus (Table 1, Figures 7(g)–7(i)). Moreover, in the experimental pigs, no statistically significant changes in the density of CGRP-immunoreactive nerve fibres were observed (Table 1, Figures 7(j)–7(l)).

**Figure 10:**
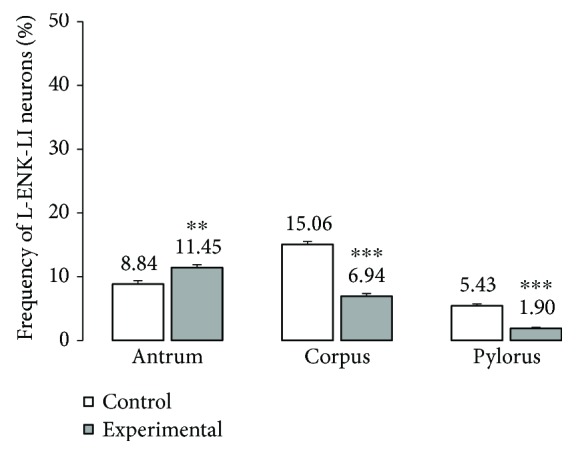
The average percentage of MP neurons immunoreactive to L-ENK. The mean of L-ENK-like immunoreactive (LENK-LI) neurons in the myenteric plexus (MP) of the antrum, corpus, and pylorus regions of the stomach in the control and streptozotocin-inducted diabetes group. Data are presented as mean ± SEM; statistically significant data (^∗∗^*P* < 0.01 and ^∗∗∗^*P* < 0.001).

**Figure 11:**
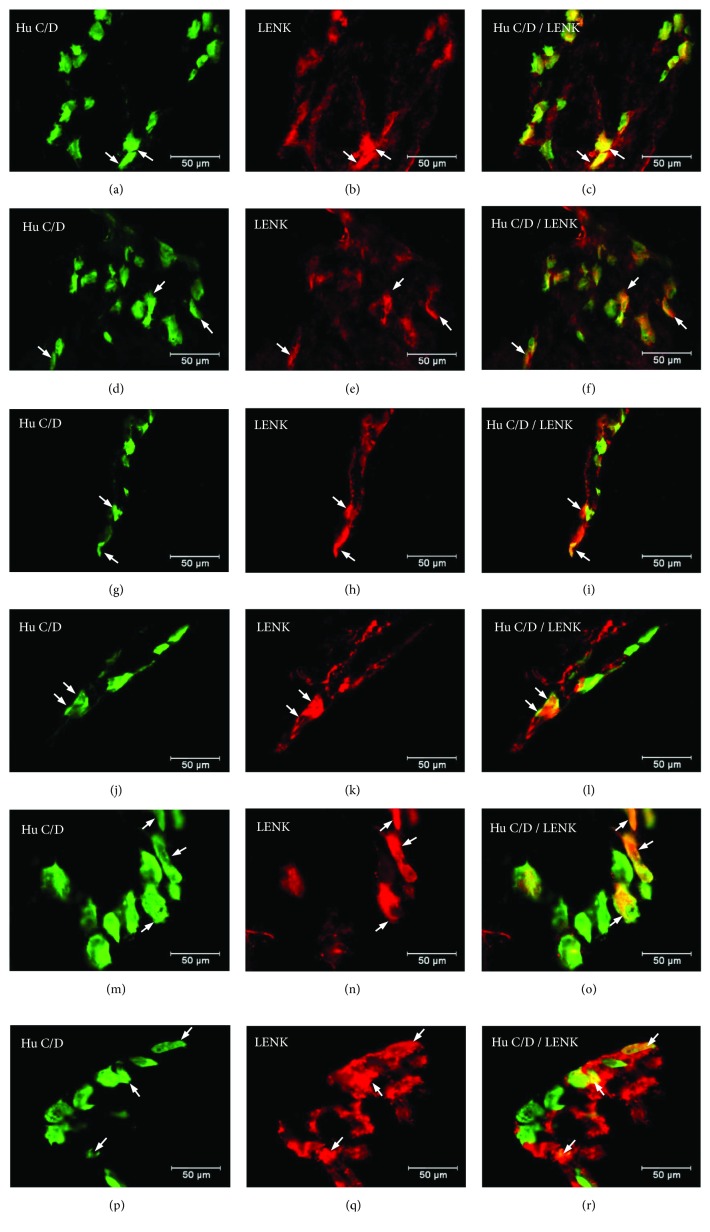
The average percentage of MP neurons immunoreactive to L-ENK. The mean of L-ENK-like immunoreactive (LENK-LI) neurons in the myenteric plexus (MP) of the antrum, corpus, and pylorus regions of the stomach in the control and streptozotocin-inducted diabetes group. Data are presented as mean ± SEM; statistically significant data (^∗∗^*P* < 0.01 and ^∗∗∗^*P* < 0.001).

Another substance studied was L-ENK. The population of neurons containing L-ENK was the least numerous among investigated peptides. In the control group, the population of L-ENK-positive neurons was the highest in the MG of the corpus (15.06 ± 0.47%) (Figure 10, Figures 11(g)–11(i)), a bit lower in the antrum (8.84 ± 0.54%) (Figure 10, Figures 11(a)–11(c)), and the lowest in the pylorus (5.43 ± 0.32%) (Figure 10, Figures 11(m)–11(o)). In turn, in the submucosal ganglia within the corpus, this value was calculated at 3.43 ± 0.40% (Figure 4, Figures 5(m)–5(o)).

Hyperglycaemia triggered the following changes in the chemical phenotyping of L-ENK-positive enteric neurons. In the MG of the antrum, an increase was noted in L-ENK-LI neurons (to 11.45 ± 0.44%) (Figure 10, Figures 11(d)–11(f)), while in the corpus, there was a decrease in L-ENK-LI neurons (to 6.94 ± 0.42%) (Figure 10, Figures 11(j)–11(l)), while in the pylorus, the number of L-ENK-immunopositive cell bodies was decreased (1.90 ± 0.16%) (Figure 10, Figures 11(p)–11(r)). In turn, in the submucosal ganglion of the corpus, the population of L-ENK-LI neurons was increased (to 6.28 ± 1.67%) (Figure 4, Figures 5(p)–5(r)).

**Figure 12:**
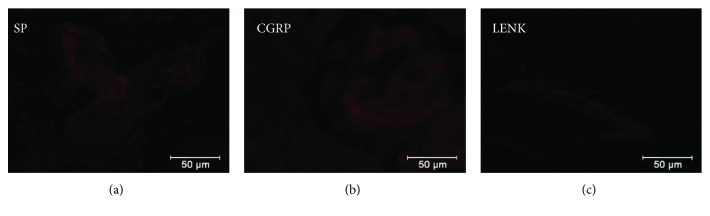
Negative controls for SP (a), CGRP (b), and LENK (c).

On the other hand, L-ENK-positive nerve fibres were visible in both the circular and submucosal muscle layers. In the control group, the density of nerve fibres within the circular muscle layer amounted to 1.63 ± 0.21 in the antrum, 3.11 ± 0.22 in the corpus, and 1.78 ± 0.15 in the pylorus (Table 1, Figures 6(m)–6(o)). In a diabetes condition, statistically significant changes were observed only in the corpus, that is, a decrease was noted in L-ENK-positive nerve fibres (to 1.47 ± 0.37) (Table 1, Figures 6(p)–6(r)). In the submucosal layer, the density of nerve fibres in the control group was as follows: 1.81 ± 0.24 in the antrum, 1.43 ± 0.09 in the corpus, and 2.25 ± 0.01 in the pylorus (Table 1, Figures 7(m)–7(o)). In the diabetic group, the density of L-ENK-containing nerves fibres changed only in a statistically insignificant manner compared to that in the control group (Figures 7(p)–7(r)). In the preabsorbtion test positive reaction was not observed (Figure 12).

## 4. Discussion

The present study showed that the porcine gastric enteric neurons, after six weeks of sustained hyperglycaemia, exhibit alterations in the chemical phenotyping of the ENS neurons situated in the porcine stomach. Hitherto, data describing chemical changes in the intramural enteric neurons in response to hyperglycaemia were limited to studies on small animal models, especially rats [12, 37, 38]. The population of the SP-IR neurons has been described in a different region of the gastrointestinal tract [20, 24, 25]. Previous studies clearly showed that the level of SP expression in the GI tract is dependent on diabetes duration. In the human stomach, after 15 years of diabetes condition, a decreased concentration of SP in Cajal cells was described [39]. However, in rats, 48 weeks after the induction of diabetes, significant changes in SP expression in both the stomach and small intestine were not observed [40]. The results obtained in the present experiment, performed on immature gilts, are different than those presented above. Namely, a statistically significant increase was observed in the number of SP-positive neurons in all investigated areas of the stomach. It is hypothesized that the observed augmentation in SP expression in diabetic pigs may be explained by the time of hyperglycaemia duration. Moreover, throughout the entire time of the experiment, all animals in the diabetic group exhibited an approx. fourfold increase in glucose serum compared to those in the control group. Additionally, none of the pigs in the diabetic group received exogenous insulin. The supplementation of exogenous insulin seems to be an important factor to restore an appropriate level of SP [41]. In turn, with respect to differentiation of SP distribution due to the region of the gastrointestinal tract, previous studies demonstrated differences between the stomach and small and large intestines, as well as among each ganglion [42–44]. For example, in diabetic rats, an increase in SP expression has been noted in the myenteric ganglia of the duodenum and colon, as well as within the ileum [12]. Moreover, we have observed an increase in the number of SP-positive nerve fibres. A similar process was also observed in other pathological conditions [20]. But it should be noted that the exact origin of these fibres (extrinsic/intrinsic) is unclear and its elucidation requires application of retrograde tracing techniques.

Furthermore, CGRP immunoreactivity was found to be significantly reduced after 8 and 12 weeks of diabetes in the myenteric and submucosal plexuses of rat ileum and colon [43, 44]. On the other hand, there is a lack of information about changes in CGRP expression in the stomach. The current study indicates that in the myenteric ganglia of the antrum, corpus, and pylorus in diabetic pigs, the expression level of CGRP was higher than the number of CGRP-LI neurons observed in the healthy group. In the submucosal ganglia in the corpus, statistically significant changes in the CGRP expression were also noted. It should be emphasized that previous studies were performed on rats using the streptozotocin diabetes model, while the current study, for the first time, used pigs as a model to focus studying on a phenotype of stomach enteric neurons. The rate of metabolism in swine is similar to humans [45]. In addition, pigs are increasingly used as experimental models in diseases with metabolic disorders [46].

The role of SP and CGRP in the gastrointestinal tract is well established [15, 16]. One of the best-known functions of SP and CGRP, not only in the gastrointestinal tract, is sensory and nociceptive information transmission [20]. Both SP and CGRP are markers of primary afferent neurons whose main role is to participate in short reflexes within the gastrointestinal wall. These neurons also take part in the transmission of nociceptive stimuli from the lamina mucosa and muscular layer of the gastrointestinal tract [30]. This observation strongly suggests that SP and CGRP may be actively involved in the pathophysiological mechanisms of abdominal pain during diabetes. This function seems to be very important because pain episodes are one of the most often disturbances in people with long-term diabetes [2]. Our studies show that an increase in the number of SP and CGRP neurons can be correlated with pain episodes. In order to fully answer of this question, further research should be carried out using analogues or agonists/antagonists of these substances to confirm the role of SP and CGRP in this process.

It should also be added that in the course of many gastrointestinal symptoms, gastrointestinal motility is impaired. Gastrointestinal symptoms including dysfunction of motor activity appear more commonly in diabetes [47]. The most important manifestation of gastrointestinal autonomic neuropathy is gastroparesis [4, 5]. The molecular mechanism underlying the above symptoms is not fully understood. The receptor for advanced glycation end products (RAGE) has recently gained attention as a potential contributor to neuropathy including autonomic neuropathy [48]. The mechanism triggering RAGE-related neurodegenerative processes is likely related to oxidative stress caused by an increase in reactive oxygen species through an NF-*κ*B-activated inflammatory pathway [49]. In the above described processes, SP and CGRP can be at least partially involved. Inflammatory and immune modulatory actions of SP are relatively well known [50]. Namely, SP is considered to be an important proinflammatory factor, which inducts cytokine release [51], as well as an agent sending information between the nervous and immune systems [50].

Leu5-enkephalin, which was also investigated in the present study, belongs to a group of opioid peptides [31]. The sources of opioid peptides in the GI tract include both endocrine cells and enteric neurons. Enkephalins are predominantly located in submucosal and myenteric neurons. They are also present in stomach muscle membranes, as well as in the muscle layer of the small and large intestines. These peptides exhibit various effects on the function of the gastrointestinal tract. Generally, they show a modulatory effect on the secretion of other peptides and are able to enhance or inhibit the contractility of particular parts of the gastrointestinal tract [17, 52, 53].

The current results, for the first time, describe changes in the expression of leu5-enkephalin in stomach enteric neurons under acute hyperglycaemia. In contrast to SP and CGRP, an increase in the number of neurons containing leu5-enkephalin in investigated areas of the stomach was not observed. With regard to myenteric ganglia, in the corpus and pylorus, a decrease in the expression of leu5-enkephalin was observed. To date, changes of leu5-enkephalin in the course of diabetes in enteric neurons have not been investigated. Till now, the expression and distribution of leu5-enkephalin were described in the human large intestine in the course of drug-resistant colitis [30]. Moreover, the distribution of opiate receptors in the gastrointestinal tract were also described [54]. It is well known that endogenous opioids have strong antinociceptive properties. Based on the current results, it can be concluded that changes in the amount of leu5-enkephalin neurons may be at least partially responsible for increasing pain reactions in the course of long-lasting hyperglycaemia in diabetes patients. Also, like in the case of CGRP and SP utilization of agonists or antagonists of opioids, receptors can provide more information concerning engagement of investigated substances in gastrointestinal complications in the course of diabetes.

## 5. Conclusion

In conclusion, the obtained results proved that hyperglycaemia causes significant changes in the neurochemical profile of the porcine stomach enteric neurons with respect to SP, CGRP, and L-ENK. It also seems that the participation of investigated substances in the development of gastric complications is significant. Undoubtedly, they are involved in the process of transmitting and modulating sensory information and may also influence the immune response, inflammatory processes, and regulate motor activity. It cannot be excluded that, in the future, research on the role of these peptides may result in the development of effective pharmacotherapy of gastrointestinal disorders. In addition, it seems that the pig is an appropriate model for studying the effect of hyperglycaemia on the neurochemical coding of enteric neurons.

## Figures and Tables

**Table 1:** SP-, CGRP-, and L-ENK-immunoreactive nerve fibres in various parts of the porcine stomach under physiological conditions (control group) and during experimentally induced diabetes (experimental group).

Stomach part	Control group			Experimental group		
	Antrum	Corpus	Pylorus	Antrum	Corpus	Pylorus
*SP*						
Circular muscle layer^a^	4.82 ± 0.16	3.65 ± 0.16	6.07 ± 0.13	7.42 ± 0.37^∗∗^	5.63 ± 0.15^∗∗∗^	10.75 ± 0.4^∗∗∗^
Submucosal/mucosal layer^a^	2.8 ± 0.28	5.19 ± 0.20	2.41 ± 0.18	6.72 ± 0.16^∗∗∗^	9.46 ± 0.41^∗∗∗^	2.89 ± 0.25
*CGRP*						
Circular muscle layer^a^	6.10 ± 0.08	6.20 ± 0.18	6.24 ± 0.33	10.44 ± 0.26^∗∗∗^	11.38 ± 0.34^∗∗∗^	8.85 ± 0.19
Submucosal/mucosal layer^a^	2.58 ± 0.12	2.08 ± 0.14	3.40 ± 0.18	2.88 ± 0.18	2.45 ± 0.09	3.14 ± 0.26
*L-ENK*						
Circular muscle layer^a^	1.63 ± 0.21	3.11 ± 0.22	1.78 ± 0.15	1.98 ± 0.12	1.47 ± 0.37^∗∗^	2.10 ± 0.24
Submucosal/mucosal layer^a^	1.81 ± 0.24	1.43 ± 0.09	2.25 ± 0.10	1.62 ± 0.51	1.58 ± 0.11	2.55 ± 0.25

Statistically significant data (^∗∗^*P* > 0.01 and ^∗∗∗^*P* > 0.001). ^a^Average number of nerve profiles per area (0.55 *μ*m^2^) studied (mean ± SEM).

## Data Availability

The data used to support the findings of this study are available from the corresponding author upon request.
